# Hyperhomocysteinemia as a Cause of Acute Ischemic Stroke in a Four-Year-Old Child: A Report of a Rare Case

**DOI:** 10.7759/cureus.46981

**Published:** 2023-10-13

**Authors:** Roopeessh Vempati, Pratyusha Ravulapalli, Sarojini Posani, Akhshaya Mahalingam, Tamara Tango, Anam Sayed Mushir Ali

**Affiliations:** 1 Internal Medicine, Gandhi Medical College and Hospital, Secunderabad, IND; 2 Cardiology, Heart and Vascular Institute, Detroit, USA; 3 Internal Medicine, Apollo Institute of Medical Sciences and Research, Hyderabad, IND; 4 Internal Medicine, Sri Devaraj Urs Medical College, Kolar, IND; 5 Pediatrics, Government Stanley Medical College, Chennai, IND; 6 Neurosurgery, Faculty of Medicine, Universitas Indonesia, Jakarta, IDN; 7 Neurology, Faculty of Medicine, Universitas Indonesia, Jakarta, IDN; 8 Internal Medicine, Faculty of Medicine, Universitas Indonesia, Jakarta, IDN; 9 Internal Medicine, Indian Institute of Medical Science and Research, Aurangabad, IND

**Keywords:** hyperhomocysteinemia, stroke, ischemic stroke, pediatric stroke, homocysteine

## Abstract

Homocysteine is a type of amino acid that isn't genetically encoded by the human body. This amino acid is capable of causing oxidative damage to the endothelial cells, leading to the onset of thrombosis. Moreover, it can also inflict harm to neurons by activating pro-apoptotic factors, causing DNA damage, and inducing oxidative stress, as observed in various animal models and cell cultures. This case report highlights a four-year-old girl who exhibited signs of an ischemic stroke. The neurological examination revealed several symptoms, including anisocoria, decreased tone, decreased power, absent reflexes on the right upper and lower extremity, and hyper extensor plantar response, accompanied by upper motor neuron seventh cranial nerve palsy. An MRI scan further confirmed the presence of an ischemic stroke in the left middle cerebral artery territory. After a thorough evaluation, the probable cause of this condition was identified as severe homocysteine elevation.

## Introduction

Homocysteine is a non-standard amino acid not coded for by our genome. Homocysteine is closely related to methionine (an essential amino acid) and cysteine (a non-essential amino acid) by the trans-sulfuration pathway. Methionine is combined with adenosine triphosphate to form S-adenosylmethionine, the principal methyl donor for all cell methylation reactions. Once S-adenosylmethionine donates the methyl group and adenosine is removed, a homocysteine molecule is formed. Thus, a new amino acid, homocysteine, is formed from another amino acid, methionine. Homocysteine can be used in two ways: 1) The re-methylation pathway: Homocysteine is regenerated into methionine by methionine synthase by adding a methyl group from N5, N10-methyltetrahydrofolate. Vitamin B12 acts as a cofactor for this pathway. 2) Homocysteine combines with serine in the presence of the enzyme cystathionine-β-synthase (with vitamin B6 as a cofactor) to form cystathionine which further produces the amino acid cysteine with intermediates alpha-ketoglutarate and succinyl Co-A. Succinyl Co-A and alpha-ketoglutarate enter the tricarboxylic acid cycle to form glucose, making homocysteine and methionine glucogenic amino acids [1].

The normal range for homocysteine levels is 5-15 μmol/L. Hyperhomocysteinemia is a medical condition where the level of homocysteine in the blood is abnormally high, above 15 μmol/L. Hyperhomocysteinemia is classified as moderate when the level is 16-30 μmol/L, intermediate when 31-100 μmol/L, and severe when above 100 μmol/L [1].

## Case presentation

A four-year-old female child arrived at the emergency room, a second born out of a consanguineous marriage and accompanied by her father, who has been her reliable informant. She presented with weakness in her right upper and lower limbs and could not speak, which was sudden in onset and progressed gradually. The child had been in good health five hours before her presentation but had then developed these concerning symptoms. Later, to the onset of symptoms, she also experienced a single episode of non-projectile, non-bilious vomiting, not associated with abdominal pain or any signs of blood. Importantly, there was no history of similar complaints in the child's past, and no history suggestive of stroke in the young people was reported in the family.

The child's birth history revealed that she was delivered via lower extremity cesarean section, weighed 2.5 kg at birth, and was admitted to the neonatal intensive care unit (NICU) due to neonatal jaundice. Her developmental history was unremarkable, with normal attainment of milestones and a developmental quotient of 100%.

**Figure 1:**
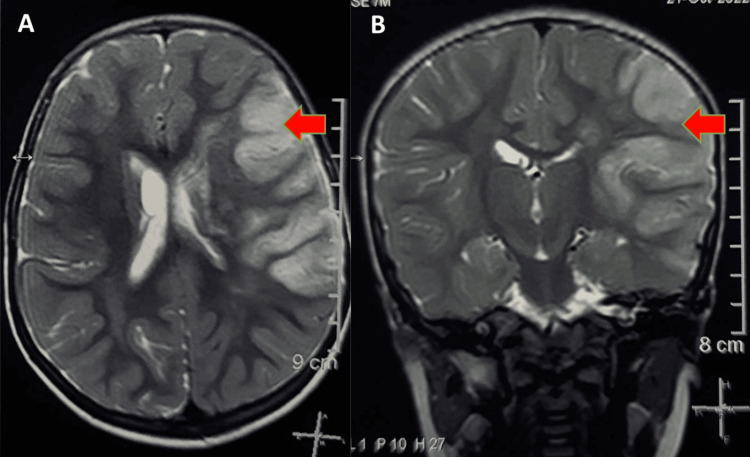
T2 weighted MRI in axial (A) and sagittal (B) section showing hyperintensity in the left middle cerebral artery distribution, that is, left temporal, parietal, frontoparietal, and parietooccipital regions.

Upon admission, the child's neurological examination revealed a Glasgow Coma Scale score of E4V2M6. Neurological examination revealed anisocoria, bilateral pupils reacting to light, decreased tone, decreased power, absent reflexes on the right upper and lower extremity and hyper extensor plantar response, and upper motor neuron seventh cranial nerve palsy. An MRI stroke protocol revealed a large T1 iso to a hypointense area T2 hyperintense area (Figure 1) in the left temporal, parietal, frontoparietal, and parietooccipital regions. This area displayed bright restriction on diffusion-weighted imaging, resulting in the effacement of adjacent sulci and pressure on the left ventricle and third ventricle, and minimal midline shift to the right, indicating acute infarcts in the left middle cerebral artery, left anterior, and posterior watershed zones. Furthermore, there was non-projection of the left middle cerebral artery from its origin on the magnetic resonance angiogram, suggesting a blockage.

At admission, the child's vital signs included a blood pressure of 110/80 mm Hg, which fell within the normal range for a four-year-old. An ECG showed sinus tachycardia. Neurological examination revealed anisocoria, bilateral pupils reacting to light, decreased tone, decreased power (two in the right upper extremity and zero in the right lower extremity out of five), absent reflexes in the right upper and lower extremities, and hyper extensor plantar responses. Moreover, there was evidence of upper motor neuron seventh cranial nerve palsy. Detailed laboratory and diagnostic workup of the patient is tabulated in Table 1.

**Table 1:** Full laboratory and diagnostic workup. MCV: Mean corpuscular volume

Parameter	Laboratory value	Physiological range
Hemoglobin (g/dl)	11.2	9.5-14
Red blood cell (mil per mm³)	4.47	4-5.5
Hematocrit (%)	34.1	30-44
MCV (fL)	81	80-100
White blood cell (1000 per mm³)	8.44	4.8-10.8
Platelet count (lakh per mm³)	4.77	1.5-4.5
Neutrophils (%)	37	40-60
Lymphocytes (%)	54	20-40
Homocysteine (uM/L)	173	<15
Vitamin B12 (ng/ml)	927	330-1236
Folic acid (ng/ml)	15	5.7-31.3
Erythrocyte sedimentation rate (mm/hr)	70 in 1st hour; 100 in 2nd hour	<10-20
Mantoux test (mm)	12	0-15
Prothrombin time (s)	15.6	11-13.5
Partial thromboplastin time (s)	30	25-35
International normalized ratio	1	=1.1
Peripheral smear	Normocytic normochromic anemia with a few hypochromic cells	
Antinuclear antibodies	Negative	
Sickling test	Negative	
2D echocardiography	Normal	
USG abdomen	Normal	
Slit lamp examination and fundoscopy	Normal	
Renal artery doppler	Normal	
Anticardiolipin antibodies (IgG & IgM)	Negative	
Factor V	Normal	
Antithrombin III	Normal	
Protein C	Normal	
Protein S	Normal	

The patient was admitted to the pediatric intensive care unit and received treatment, including furosemide 1 mg/kg/day intravenously (IV), and labetalol 0.3 mg/kg/hr IV to control the blood pressure, stopped after blood pressure normalized. Later, hourly blood pressure monitoring and findings were within the physiological range. In addition, injections of mannitol 2 ml/kg were given IV in the first two days, low-molecular-weight heparin 1 mg/kg/dose subcutaneously every 12 hours for the first three days, then 1 mg/kg/day for 14 days, and aspirin tablet 5 mg/kg/day continued toward the end of hospitalization, and syrups of pyridoxine, methylcobalamin, and folic acid were also added.

## Discussion

**Figure 2:**
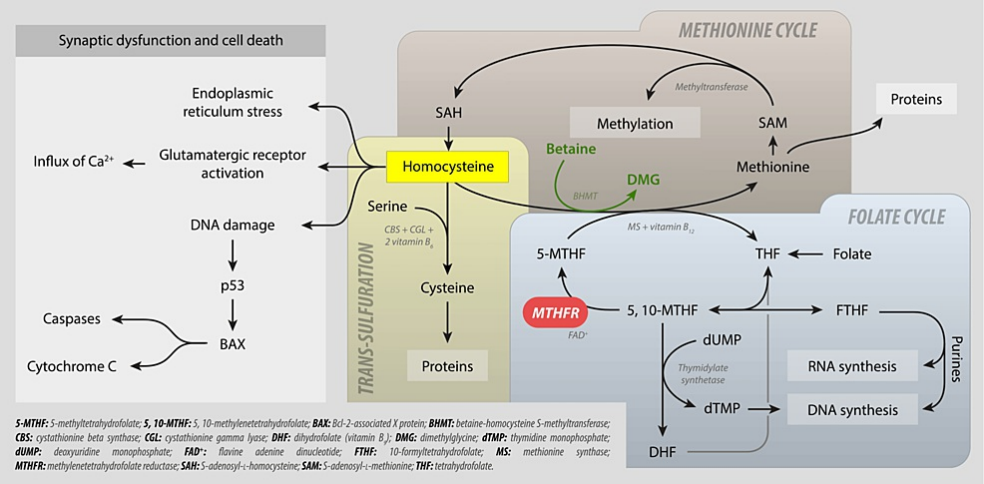
Homocysteine metabolism Picture credits: Wikipedia

Homocysteine metabolism is depicted in Figure 2. Autosomal recessive defects in homocysteine metabolism can cause hyperhomocysteinemia. There are various enzymes involved in the condition of elevated homocysteine levels, including 5,10-methylenetetrahydrofolate reductase, methionine synthase, and cystathionine-β-synthase (in cases of classic homocystinuria) [2]. Moreover, insufficient intake of vitamins B12, B6, and B9 through diet can also lead to this condition. Various factors may be linked to slightly increased levels of homocysteine, such as kidney and thyroid problems, cancer, psoriasis, diabetes, certain medications, alcohol, tobacco, coffee, advanced age, and menopause. Furthermore, a rise in serum creatinine levels can cause an increase in fasting total homocysteine [1].

Studies indicate that elevated levels of homocysteine in the bloodstream can promote blood clot formation, increasing the risk of stroke and cardiovascular disease. Research has found a connection between the amount of homocysteine in the blood and the occurrence of coronary, peripheral vascular, and carotid diseases. A systematic review of 12 randomized controlled trials examined the effectiveness of interventions in reducing homocysteine levels in 47,429 subjects. Regrettably, interventions aimed at reducing homocysteine levels did not significantly impact heart attack, stroke, or mortality rates when compared to a placebo. Research suggests that excessive levels of homocysteine may harm endothelial cells, decrease blood vessel flexibility, and interfere with hemostasis. Homocysteine has also been linked to neuronal damage through the promotion of oxidative stress, DNA damage, and activation of pro-apoptotic factors in cell cultures and animal models. The compound homocysteine may act as an excitatory agonist on N-methyl-D-aspartate (NMDA) subtype glutamate receptors, with recent evidence suggesting the involvement of NMDA modulatory sites in its neurotransmitter action. It has also been shown that homocysteine, besides acting as a partial agonist at glutamate receptors, also acts as a partial antagonist of the glycine co-agonist site of the NMDA receptor [1].

Children experiencing a stroke or cerebrovascular accident is a rare occurrence. The incidence range of combined ischemic and hemorrhagic pediatric stroke is 1.2 to 13 cases per 100,000 children under 18 years old. Boys are more prone to stroke compared to girls, even when considering the causes, such as trauma [3].

In a study of Dutch children aged 0 to 18 with ischemic stroke, 18% of the 45 patients had hyperhomocysteinemia. The study aimed to determine the possible relationship between moderate hyperhomocysteinemia and ischemic stroke [4].

Children with ischemic stroke typically show signs of a localized neurological issue, with hemiplegia being the most common manifestation in about 94% of cases. Additionally, homocystinuria may lead to acute ischemic stroke (AIS), particularly if accompanied by lens dislocation, mental retardation, and in some cases, pectus excavatum.

AIS may also cause changes in mental state, headache, seizure, fever, and prodromal symptoms. Children's stroke symptoms vary depending on their age. Newborns may experience focal seizures, lethargy, and apnea. Toddlers may exhibit a range of symptoms, including a decline in general condition, increased crying and sleepiness, irritability, feeding problems, vomiting, and sepsis-like symptoms with cold extremities. Older children may develop specific neurological deficits like hemiparesis, language difficulties (such as aphasia), speech problems, visual deficits, and headaches [3].

In order to diagnose this case, a comprehensive list of possible causes was considered and systematically ruled out. First, congenital heart diseases, such as cyanotic lesions, cardiomyopathies, rheumatic heart disease, and valvular issues, were eliminated through a normal 2D echocardiogram. Possible sources of embolic stroke, such as patent foramen oval and septal defects, were also investigated. Sickle cell disease, a common cause of pediatric stroke, was ruled out due to a negative sickening test result. Antithrombin 3 deficiency was eliminated as antithrombin levels were found to be within the normal range. Protein C and protein S deficiencies were also excluded with normal levels, while a coagulation profile, including prothrombin time, partial thromboplastin time, and international normalized ratio, was within normal limits to dismiss clotting factor deficiencies. Renal and liver pathologies, including nephrotic syndrome, were considered but ultimately excluded through a normal abdominal ultrasound. Folic acid and vitamin B12 deficiencies were also ruled out as causes, as serological levels and peripheral smear findings were normal. Tuberculous etiology was assessed but deemed unlikely with a negative Mantoux test, especially in the context of its endemicity in the Indian population. Antiphospholipid antibody syndrome was eliminated as a possibility through negative anticardiolipin IgG and IgM tests. Lastly, arterio-venous malformation was ruled out based on negative MRI findings, and congenital renal artery stenosis was eliminated with a normal renal artery Doppler. This thorough process of elimination has allowed for a more focused approach to determining the underlying cause of the case [3].

Pediatric stroke has a high rate of morbidity and mortality. Around 10-25% of children who experience a stroke will unfortunately pass away, while up to 25% may experience a recurrence. Additionally, up to 66% may suffer from ongoing neurological deficits or develop issues such as seizure disorders, learning difficulties, or developmental problems. Due to the fact that symptoms of pediatric stroke can be non-specific and subtle, there is a greater risk of misdiagnosis or delayed diagnosis. It is important to recognize that children with strokes may display different symptoms than adults and often have unique risk factors. By acknowledging these differences, we can improve outcomes for children affected by stroke [3].

## Conclusions

The incidence of pediatric stroke is very rare and therefore, it is imperative to conduct a comprehensive investigation into the root cause. To ensure a thorough evaluation process, it is recommended to include a serum homocysteine test. The combination of vitamin B6, vitamin B12, and folic acid can be of great assistance in reducing serum homocysteine levels and is a significant aspect of managing the condition. Following the release, the patient was monitored regularly and exhibited a noticeable improvement in their neurological symptoms, with only a minor residual neurological deficit.
